# Research progress on Alzheimer’s disease vaccines: from Aβ-targeted approaches to clinical translation

**DOI:** 10.3389/fragi.2026.1794820

**Published:** 2026-04-08

**Authors:** Yan Han, Gaomei Luo, Yanhao Huang, Zhicheng Cai, Wei Su, Xuejun Kuang, Lijuan Huang, Li Wei, Fenghuang Ming, Ziqi He, Zimeng Yang, Jingqi Wang, Wei Jiang, Chanchan Xiao, Qingbing Hu, Jianhui Yan

**Affiliations:** 1 Affiliated Hospital of Xiangnan University, Chenzhou, Hunan, China; 2 The First Affiliated Hospital, Jinan University, Guangzhou, Guangdong, China; 3 Xiangnan University, Chenzhou, Hunan, China; 4 School of Nursing, Xiangnan University, Chenzhou, Hunan, China; 5 Clinical College of Xiangnan University, Chenzhou, Hunan, China

**Keywords:** Alzheimer’s disease, clinical translation, immunotherapy, vaccine, β-amyloid protein

## Abstract

**Background:**

Alzheimer’s disease (AD) is a prevalent neurodegenerative disorder characterized by progressive cognitive impairment, with the β-amyloid protein (Aβ) aggregation as a core pathological driver. As global aging intensifies, AD poses a severe public health burden, highlighting the urgency of developing effective immunotherapies. This review aims to systematically summarize the research progress of Aβ-targeted AD vaccines, from first-generation approaches to next-generation strategies, and discuss key challenges and future directions for clinical translation.

**Methods:**

A comprehensive literature search was conducted across PubMed, Web of Science, the Cochrane Library, EMBASE, and Google Scholar up to 14 November 2025. Relevant studies were selected using predefined eligibility criteria, focusing on Aβ-targeted AD vaccines’ development, mechanisms, preclinical efficacy, and clinical outcomes. Review articles and meta-analyses were included, while case reports and non-Aβ-targeted studies were excluded. Data extraction and synthesis focused on vaccine strategies, immune mechanisms, and translational challenges.

**Results:**

First-generation Aβ vaccine (e.g., AN-1792) showed preclinical promise but failed clinically due to autoimmune complications. Next-generation vaccines, including peptide/epitope vaccines, DNA vaccines, viral vector vaccines, and protein self-assembling vaccines, have been developed to induce protective Th2-biased immune responses while avoiding harmful T-cell reactions. Preclinical studies demonstrate reduced Aβ deposition and improved cognitive function, with several candidates advancing to clinical trials showing favorable safety and immunogenicity. Key mechanisms include Fc receptor-mediated phagocytosis, antibody-mediated fibril disaggregation, and the peripheral Aβ sink effect.

**Conclusion:**

Aβ-targeted AD vaccines have evolved toward safer and more effective designs, with multiple strategies showing translational potential. Challenges remain, including blood-brain barrier (BBB) penetration, immune response modulation, and defining optimal therapeutic time windows. Future research should focus on personalized vaccines, combination therapies, and novel antigen delivery platforms to fully realize the clinical potential of AD immunotherapies.

## 1 Introduction

**FIGURE 1:**
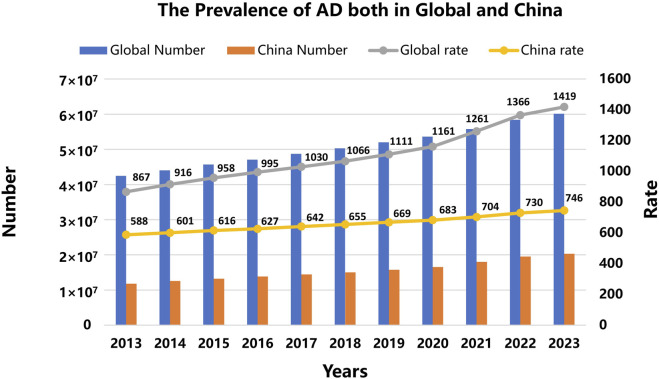
The prevalence of AD in global and Chinese populations from 2013 to 2023. Fueled by advancements in medical technology and societal progress, human life expectancy has undergone a consistent increase, accompanied by a steady rise in the proportion of the elderly population over time—trends that collectively underscore the global demographic shift toward aging. Against this epidemiological backdrop, the absolute number of patients with AD and its population prevalence have exhibited a gradual upward trajectory both globally and within China. Consequently, the prevention of AD has emerged as an increasingly salient research priority in the realms of clinical medicine and public health. (see https://www.healthdata.org/research-analysis/gbd (accessed on 1 November 2025) for more details).

Alzheimer’s disease (AD) represents the most prevalent form of senile dementia, clinically characterized by progressive cognitive decline, memory impairment, deficits in judgment and cognition, along with personality alterations. Statistical data indicate over 60 million AD patients worldwide. In China, AD patients exceed 20 million, imposing a significant economic burden on society and families (Figure 1).

The primary pathological features of AD include extracellular senile plaques (SP), intracellular neurofibrillary tangles (NFTs), and granulovacuolar degeneration. The principal component of SP is the β-amyloid protein (Aβ), a 39–42 amino acid residue polypeptide generated through sequential cleavage of amyloid precursor protein (app) by β- and γ-secretases. Aβ possesses self-aggregation capability, enabling its extracellular assembly into oligomers, progression to fibrils, and ultimate formation into amyloid deposits. Research suggests that aberrant aggregation of Aβ constitutes the initial and most critical step in the entire neuropathological process of AD. This phenomenon not only directly exerts neurotoxicity leading to neuronal dysfunction and degeneration, but may also trigger hyperphosphorylation of tau protein and subsequent formation of NFTs (Selkoe, 2001).

Given the central role of Aβ in AD pathogenesis, researchers proposed the “Amyloid Cascade Hypothesis,” which has laid the theoretical groundwork for AD immunotherapy. In 1999, Schenk et al. (1999) first reported that active immunization with Aβ42 in PDAPP transgenic mice successfully prevented and reduced cerebral Aβ deposition, pioneering a new Frontier in AD immunotherapy. This groundbreaking advancement has rapidly propelled AD vaccine research from animal experiments to clinical trials. However, the development of AD vaccines has not been straightforward. The first-generation vaccine AN-1792 was terminated during its Phase II clinical trial due to induction of autoimmune meningoencephalitis, prompting researchers to deeply explore the mechanism of action and safety concerns of AD vaccines. This has led to the development of multiple next-generation vaccine strategies.

This article systematically reviews research progress on AD vaccines, comprehensively summarizing the current landscape from lessons learned with first-generation vaccines to innovations in next-generation approaches, and from mechanism of action to clinical translation. It further provides perspectives on future research directions.

## 2 Results

### 2.1 Research strategy

The following databases were investigated in an attempt to identify all the relevant studies published on PubMed, Web of Science, the Cochrane Library, EMBASE, and Google Scholar. The literature search was conducted up to 14 November 2025. The topic search terms used were as follows: #1—“Alzheimer’s disease” OR “AD”; #2—“β-amyloid” OR “beta-amyloid” OR “Aβ”; #3—“vaccine” OR “vaccination” OR “active immunization”; and #4—#1 AND #2 AND #3. During the search process, the word forms and logical operators were adjusted in accordance with the retrieval rules of each database to improve search comprehensiveness.

### 2.2 Eligibility criteria

We included published articles reporting AD and corelated vaccines. Only the studies published in English focusing on the development, mechanism of action, preclinical experiments, or clinical research of Aβ-targeted AD vaccines were eligible for inclusion. Review articles and meta-analyses were included. Case reports, case series, and opinion articles were excluded.

To evaluate the mechanisms underlying Aβ-targeted AD vaccines, the vaccination mechanisms, construction strategies, and future research directions were investigated individually. Studies on non-Aβ-targeted AD vaccines (e.g., vaccines targeting only a single non-Aβ target such as tau protein or neuroinflammation), literature that did not clearly elaborate on the vaccine design, immunization strategy, or lacked core experimental data/clinical outcomes, and literature with obvious errors in research data, logical contradictions, or methodological flaws that could not be verified were excluded.

### 2.3 Study selection

**FIGURE 2:**
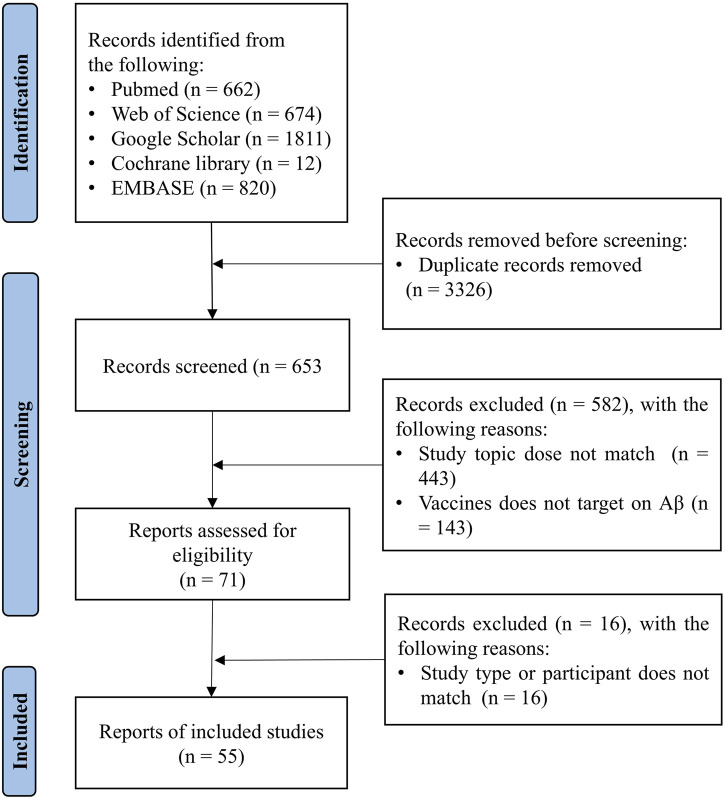
Study selection process for Aβ-targeted Alzheimer’s disease vaccines.

After manually removing duplicates, the titles and abstracts of the articles identified through the initial search were first screened. The full texts of the relevant articles were examined for the inclusion and exclusion criteria (Figure 2). After screening the abstracts, the full texts of the articles were assessed for eligibility and were selected or rejected for inclusion in the review. Any discordant results were discussed in a consensus meeting.

## 3 The emergence and initial exploration of Aβ immunotherapy

### 3.1 Animal experimental achievements of AN-1792 vaccine

In 1999, Schenk et al. (1999) first reported research results on immunizing PDAPP transgenic mice with synthetic human Aβ42 combined with Freund’s adjuvant. PDAPP mice are transgenic mice overexpressing mutant human APP genes, which spontaneously develop AD-like pathological changes including cerebral Aβ deposition, senile plaque formation, and cognitive impairment as they age. The study found that immunoprophylaxis in young PDAPP mice before Aβ deposition occurs can effectively prevent the formation of cerebral Aβ deposits; Immunotherapy administered to aged PDAPP mice with established Aβ deposition significantly reduced existing Aβ plaque burden. This breakthrough finding indicates that Aβ immunotherapy holds not only preventive potential but may also exert therapeutic effects on pre-existing pathological alterations.

Subsequently, multiple research teams validated the efficacy of Aβ immunotherapy across diverse AD transgenic mouse models. Janus et al. (2000) Immunized transgenic mice expressing mutant human APP with Aβ42. Morris water maze testing demonstrated that active immunization strategies significantly ameliorated behavioral deficits in these mice. Morgan et al. (2000) demonstrated in hAPP + PS1 transgenic mice that Aβ42 immunization not only reduces cerebral Aβ deposition but also prevents memory loss while improving learning and memory capabilities. Notably, in certain animal models, although Aβ immunotherapy exhibits limited efficacy against pre-existing amyloid deposition, it still ameliorates behavioral impairments in mice. This suggests that Aβ immunotherapy may function by clearing toxic soluble Aβ oligomers rather than solely targeting insoluble deposits. The success of animal experiments has established a robust foundation for advancing Aβ immunotherapy toward clinical applications, fostering substantial anticipation for this novel AD therapeutic strategy.

### 3.2 Setbacks and lessons from the AN-1792 clinical trial

Encouraged by success in animal studies, the Aβ vaccine AN-1792 (synthetic human Aβ42 with QS-21 adjuvant) developed by the U.S. company Elan rapidly entered clinical trials in 2001. Phase I clinical trial results demonstrated good tolerability across various doses and induced robust humoral immune responses in a significant proportion of patients (Hock et al., 2003). This allowed AN-1792 to advance to a Phase II clinical trial.

However, in 2002, among 360 patients participating in the Phase II clinical trial, 15 subsequently developed non-bacterial inflammation of the central nervous system (autoimmune meningoencephalitis), with an additional 2 cases experiencing localized ischemic stroke. These adverse events led to the premature termination of this clinical trial (Check, 2002). Autopsy findings from deceased cases revealed both promise and concerns: On one hand, AD patients with robust immune responses showed significant reduction in amyloid deposition plaques in the neocortex. For instance, nearly no plaques were observed in the frontal lobe, whereas unimmunized control AD patients exhibited approximately 100 plaques/mm^2^ in this region. This demonstrates the vaccine’s efficacy in clearing Aβ deposition; On the other hand, inflammatory responses primarily occurred in areas with severe cerebral amyloid angiopathy (CAA). Lymphocytes infiltrated the meninges by crossing the damaged blood-brain barrier (BBB), triggering autoimmune meningoencephalitis (Nicoll et al., 2003). Further analysis revealed that the T-cell immune response elicited by AN-1792 was the primary cause of aseptic meningoencephalitis. Aβ42 contains a B-cell epitope (region 1-15) and a T-cell epitope (regions 16-33). Monsonego et al. (2003), Lemere (2013) when full-length Aβ42 is used for immunization, it induces anti-Aβ antibody production but simultaneously activates Aβ-specific T cells. These activated T cells may cross the compromised BBB, enter the central nervous system, recognize Aβ deposition in vascular walls and brain parenchyma, and trigger inflammatory responses (Orgogozo et al., 2003). The setback in the AN-1792 clinical trial led to the realization that an ideal AD vaccine should induce sufficient protective antibodies while avoiding harmful T-cell immune responses. This understanding has driven the research and development of next-generation AD vaccines.

### 3.3 Mechanism of action of Aβ immunotherapy

In the past, three main hypotheses existed regarding the mechanism of action of Aβ immunotherapy: Fc receptor-mediated microglial phagocytosis, antibody-mediated disaggregation of amyloid fibrils, and the peripheral Aβ sink hypothesis. In recent years, a growing body of research has supported Fc receptor-mediated microglial phagocytosis as the primary mechanism of action for Aβ immunotherapy, while the other two hypotheses have become increasingly considered outdated. The following section will systematically introduce the content of these three hypotheses and related research findings.

#### 3.3.1 Fc receptor-mediated microglial phagocytosis

Schenk et al. first proposed this mechanism (Schenk et al., 1999), suggesting that after anti-Aβ antibodies enter the central nervous system, their Fc fragments bind to Fc receptors on activated microglia, thereby mediating phagocytosis and clearance of Aβ deposition. Bard et al.'s study (Bacskai et al., 2001) provided strong support for this mechanism. They found that continuous intraperitoneal injection of anti-Aβ monoclonal antibodies into PDAPP mice activated microglia to clear amyloid plaques, and this activation was induced by the Fc fragment rather than the F (ab')2 fragment of the monoclonal antibody. *In vivo* multiphoton imaging technology directly revealed the dynamic process whereby microglia surround Aβ deposits and progressively clear them following antibody therapy (Bacskai et al., 2001). Donanemab is a monoclonal antibody that specifically binds to Aβ plaques modified by pyroglutamate in AD. By targeting N3pG Aβ, it enhances microglial activity, thereby reducing the level of amyloid deposition in the brain (Jayaprakash and Elumalai, 2025). Lecanemab is an antibody with high affinity for soluble Aβ protofibrils. By clearing these neurotoxic intermediate aggregates, it not only inhibits the formation of new amyloid plaques but also promotes the clearance of existing plaques through mechanisms such as microglial activation, thereby exerting its therapeutic effect in the early stages of AD. Giulia Albertini et al. administered Lecanemab to a mouse model of AD xenografted with human microglia and observed effective clearance of amyloid plaques. This therapeutic effect was found to be critically dependent on the presence of microglia and the involvement of Fc effector function. They further identified Osteopontin as a key factor induced by Lecanemab treatment and demonstrated its active role in promoting Aβ clearance (Kwon and Koh, 2025; Albertini et al., 2026). Both have received full approval from the U.S. Food and Drug Administration. Synthesizing the available evidence, particularly clinical data from successful passive immunotherapies, Fc receptor-mediated microglial phagocytosis is considered the primary mechanism of action for Aβ immunotherapy, including active vaccines.

#### 3.3.2 Antibody-mediated disaggregation of amyloid fibrils

From a protein folding perspective, Solomon et al. proposed that certain anti-Aβ antibodies function like molecular chaperones, recognizing amyloid folding initiation sites and reverting fibrillar Aβ to non-fibrillar conformations (Solomon et al., 1996). Their *in vitro* experiments demonstrated that monoclonal antibody AMY-33, which targets the Aβ fragment corresponding to residues 1-28 of the Aβ peptide, can disaggregate pre-formed Aβ fibrils. However, as academic research in this area has progressed, this hypothesis has been gradually reshaped. Antibody-mediated disaggregation of amyloid fibrils is, in fact, a mechanism that acts in synergy with microglia. The Fab domain of the antibody binds with high affinity to soluble Aβ protofibrils. This binding not only neutralizes the more toxic soluble aggregates but may also prevent the further extension and maturation of soluble protofibrils into insoluble plaques through steric hindrance or conformational interference (Valls-Comamala et al., 2017). Concurrently, the Fc domain of the antibody can be engaged by Fc receptors on microglia, thereby activating microglia and enhancing their phagocytic capacity (Albertini et al., 2026). Furthermore, relevant studies indicate that antibody binding to Aβ can also promote the phagocytic degradation of Aβ by microglia through the activation of the classical complement pathway in the early stages of complement activation. However, it is noteworthy that complete complement activation would generate the neurotoxic membrane attack complex C5b-9 (Loeffler, 2023). A thorough understanding of the synergistic interplay between these two mechanisms will contribute to the future advancement of immunotherapy in the treatment of AD.

#### 3.3.3 Peripheral Aβ sink hypothesis

DeMattos et al. (2001) proposed a fundamentally distinct mechanism: the peripheral Aβ sink hypothesis. Following injection of the anti-Aβ monoclonal antibody m266 into PDAPP mice, plasma Aβ levels surged approximately 1000-fold. Given that Aβ is primarily produced in the brain, they posited that peripheral antibodies binding plasma Aβ alter the Aβ equilibrium between the central nervous system and periphery. This shift promotes Aβ efflux from the brain into peripheral blood, thereby reducing cerebral Aβ deposition. This mechanism does not require antibodies to cross the BBB; instead, it functions by altering Aβ distribution. Studies supporting this view demonstrate that soluble Aβ can be transported relatively freely across the BBB between the central nervous system and peripheral circulation (Shibata et al., 2000). However, Henderson et al. (2014), using a modified version of the amyloid-degrading enzyme neprilysin, achieved sustained clearance of Aβ from the peripheral blood of rodents and non-human primates. Despite this significant reduction in peripheral Aβ levels, they found that it did not affect Aβ levels within the central nervous system. The results of this experiment indicated that the peripheral Aβ sink hypothesis is not valid. A deeper understanding of these three mechanisms successfully demonstrates the superiority of the Fc receptor-mediated microglial phagocytosis hypothesis, providing important guidance for optimizing vaccine strategies for AD.

## 4 Strategies and development of next-generation active immunization vaccines

Immunotherapeutic strategies for AD can be broadly categorized into passive and active immunotherapy. Passive immunotherapy involves the direct administration of anti-Aβ antibodies that target specific antigenic epitopes on Aβ, offering enhanced efficiency in directly clearing β-amyloid. This method offers high specificity in target recognition, allows for the selection of different IgG subtypes depending on the disease, and has a rapid onset of action. However, this approach is associated with high costs and the potential for cross-reactivity due to the high affinity of these antibodies (Sha et al., 2026). The aforementioned monoclonal antibody AMY-33, as well as emerging agents such as Lecanemab and Donanemab, fall within the scope of passive immunotherapy (Jin et al., 2024).

In contrast, active immunotherapy induces the patient’s own cells to participate in the humoral immune response to achieve a therapeutic effect, primarily in the form of vaccines. Its advantages include longer duration of sustained antibody titers, as well as lower costs and a reduced risk of side effects. Its main disadvantages are a slow onset of action and the potential for inducing immune tolerance (Jin et al., 2024). As the focal point of this article, vaccines will be discussed in detail in the following sections.

### 4.1 Classification of active immunization vaccines based on antigen form

#### 4.1.1 Protein/peptide epitope vaccines

To avoid the T cell-mediated autoimmune responses observed in AN-1792, re-searchers began focusing on shorter Aβ fragments as immunogens. These fragments retain B-cell epitopes while eliminating T-cell epitopes.

Studies indicate that the B-cell antigenic epitopes of Aβ primarily reside in the 1-15 region, while T-cell epitopes are located in the 16-33 region (Monsonego et al., 2003; Lemere, 2013). Therefore, using Aβ1-15 or similar short peptides as immunogens is expected to induce anti-Aβ anti-body production while avoiding activation of Aβ-specific T cells. Moretto et al. (2007) reported the expression in *Escherichia coli* of a recombinant fusion protein, 4/8Aβ(1-15)-trx, which was constructed by tandem multimerization of the immunodominant B-cell epitope Aβ1-15 within the active-site loop of bacterial thioredoxin. Agadjanyan et al. (2005) synthesized the multi-epitope vaccine PADRE-Aβ1-15 by combining Aβ′s B-cell epitope Aβ1-15 with the universal T-helper cell epitope PADRE. Immunization of BALB/c mice with this vaccine elicited specific anti-Aβ antibodies accompanied by a Th2-type immune response, with no detectable T-cell proliferative response against Aβ. Serum antibodies generated by this epitope vaccine demonstrated binding capability to Aβ plaques in the brain, indicating therapeutic potential. Rossi et al. (2009) employed glycine modification techniques to alter Aβ1-16, Aβ13-28, and Aβ25-42 peptides. This approach preserved immunogenicity against Aβ while modifying epitopes responsible for intrinsic Th1-type immune responses, thereby circumventing harmful T-cell reactions.

In addition to linear epitopes, the AFFITOPE technology based on conformational mimicry has also shown potential. The AFFITOPE technology utilizes short peptides as antigenic components that differ from the native Aβ sequence but mimic aspects of its three-dimensional structure. AD02, a product developed using this technology, mimics the structure of the N-terminus of the Aβ protein and demonstrated a generally favorable tolerability profile in a Phase 2 study involving patients with early-stage AD. However, its development was discontinued due to a lack of significant therapeutic efficacy (Mandler et al., 2015; Schneeberger et al., 2015). Nonetheless, the safety and tolerability exhibited by the AD02 vaccine may suggest that designing vaccines through more refined molecular mimicry represents a viable approach to achieving safety.

Based on these findings, several pharmaceutical companies have developed a variety of short peptide vaccines designed with the principle of avoiding T-cell epitopes. These vaccines target diverse epitopes, including the N-terminal linear epitope, the C-terminal region, and specific conformational epitopes of Aβ.

CAD106, developed by Novartis, employs repeated Aβ1-6 sequences conjugated to Qβ virus-like particles (VLPs) (Farlow et al., 2015). Preclinical studies demonstrated that CAD106 induces hightiter anti-Aβ antibodies in non-human primates, predominantly eliciting a Th2-type response. Initial clinical trials indicated that CAD106 exhibits favorable safety and immunogenicity profiles in AD patients. However, in the Phase 2b clinical trial, the study found a high incidence of Amyloid-Related Imaging Abnormalities, which ultimately halted the further development of CAD-106 (Vandenberghe et al., 2017). CAD-106, as the first second-generation AD vaccine to enter human trials, served as a benchmark. It not only demonstrated the feasibility of AD vaccines but also provided valuable lessons for subsequent AD vaccine research and development.

ACC-001 was co-developed by Elan and Wyeth. It utilizes a mutated diphtheria toxin (CRM197) as the carrier protein conjugated with the N-terminal Aβ peptide sequence, using QS21 as the adjuvant (Hull et al., 2017). This vaccine completed Phase 2 clinical trials. Although it demonstrated an antibody response and an acceptable safety profile in patients with early AD, its subsequent development was halted due to a poor-quality immune response and insufficient efficacy (van Dyck et al., 2016).

ABvac40, developed by AraclonBiotech, Aβ target is Aβ33-40. It is conjugated to a helper T-cell carrier protein and formulated in a Th2-skewed adjuvant. In the phase II clinical trial, this vaccine not only induced a robust immune response and demonstrated its potential for the long-term treatment of AD, but also exhibited favorable safety and tolerability profiles without unexpected safety concerns (Pascual-Lucas et al., 2025).

UB-311, developed by United Neuroscience, is an active immunotherapeutic agent targeting Aβ1-14 based on synthetic peptides, consisting of the Aβ1-14 B-cell epitope conjugated with two proprietary synthetic helper T-cell peptide epitopes, and formulated with a Th2-biased delivery system. The evaluation of UB-311 in the Phase 2a clinical trial demonstrated its favorable safety, tolerability, and immunogenicity (Yu et al., 2023).

ALZ-101, developed by Alzinova AB, is a vaccine composed of stabilized oligomeric Aβ42. In a Phase 1b clinical trial, it successfully generated a specific humoral immune response against Aβ oligomers and demonstrated a favorable safety profile in patients with mild AD (Zetterberg et al., 2025). A Phase 2 clinical trial is currently in active preparation.

These vaccines share the common feature of utilizing short Aβ fragments, aiming to induce protective antibody responses while minimizing adverse T-cell responses.

#### 4.1.2 DNA vaccines

DNA vaccines represent another significant direction in AD vaccine research. Compared to traditional protein/peptide vaccines, DNA vaccines offer multiple advantages: ease of large-scale production; simple handling procedures; low manufacturing costs; high safety profile; elimination of viral infection and transformation risks; ability to induce potent immune responses; non-integration into the host genome; and absence of anti-DNA autoimmune response induction (Okura and Matsumoto, 2009). These characteristics make DNA vaccines particularly well-suited for chronic conditions like AD that require long-term therapeutic intervention.

AD DNA vaccines represent an effective therapeutic approach that delivers target antigen-encoding DNA into the body either via vector-based delivery or as naked DNA directly. They induce host cells to express the corresponding antigen, ultimately eliciting the production of specific antibodies. To enhance the antibody expression level, researchers typically use molecular adjuvants in combination.

Okura et al. (2006) pioneered cloning the Aβ1-42 gene into a eukaryotic expression vector, incorporating an Ig signal peptide and Ig Fc fragment to construct an Aβ epitope DNA vaccine. Immunization produced Aβ-specific antibodies with a predominant Th2-type immune response. Ghochikyan et al. (2003) designed a combined DNA vaccine containing DNA sequences encoding human Aβ1-42 and IL-4 as a molecular adjuvant. Immunized mice developed Aβ-specific antibodies, primarily of the IgG1 subtype (indicative of a Th2-type response). This indicates that cytokines, as molecular adjuvants, can effectively modulate the type of immune response, biasing it toward the protective Th2-type. Movsesyan et al. (2008a) designed a novel DNA epitope vaccine containing three tandem repeats of Aβ1-11 and the universal T-cell epitope PADRE, while incorporating macrophage-derived chemokine (MDC/CCL22) as a molecular adjuvant. Following immunization of 3xTg-AD model mice, this vaccine elicited a robust Th2-type immune response and generated high-titer Aβ-specific antibodies. Movsesyan et al. (2008b) demonstrated that the DNA epitope vaccine 3Aβ1-11-PADRE-3C3d, constructed using complement C3d, significantly elevated antibody levels while shifting the immune response toward a Th2-type. It has been reported that other cytokines, such as IL-10 and granulocyte-macrophage colony-stimulating factor, have also been attempted for use as molecular adjuvants to regulate the type and intensity of immune responses. AV-1959D, a DNA vaccine targeting the N-terminal epitope of Aβ, has demonstrated efficacy and immunogenicity in a mouse model of AD (Petrushina et al., 2020). As an emerging therapeutic agent, it is currently in an active Phase 1 clinical trial.

### 4.2 Innovations in vaccine delivery and display platforms

**FIGURE 3:**
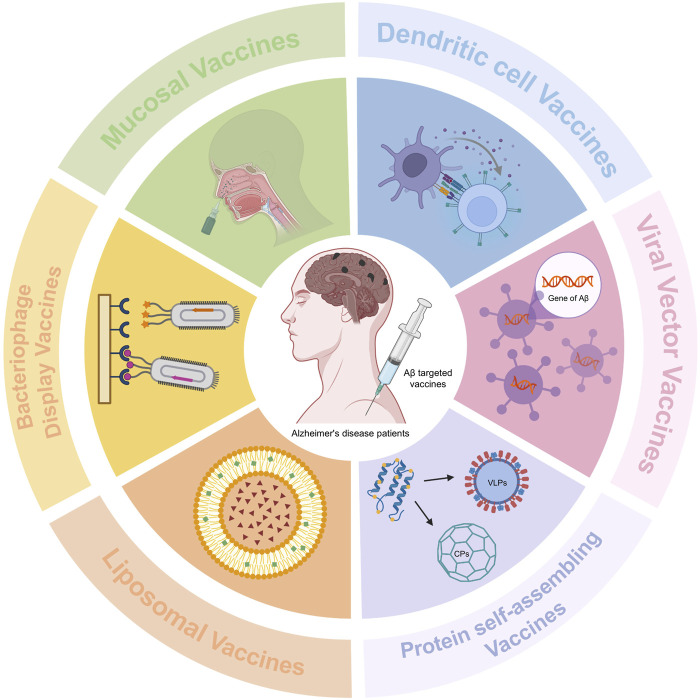
Mechanisms of Action of Innovative Vaccine Delivery and Display Platforms. In the process of vaccine development, beyond the form of the antigen itself, the vaccine delivery or display system represents another significant and promising area of innovation. This figure illustrates the mechanisms of action for several innovative vaccine delivery or display systems. (1) DC Vaccines: An experimental immunotherapy that utilizes a patient’s own specially treated DCs to induce the production of antibodies against Aβ. This approach aims to safely clear toxic protein deposits in the brain. (2) Viral Vector Vaccines: These vaccines employ recombinant viruses to deliver the gene encoding Aβ into host cells. This enables the sustained expression of the Aβ antigen *in vivo*, thereby inducing the production of anti-Aβ antibodies and leading to improved cognitive function. Notably, oral formulations offer advantages by eliminating the need for adjuvants and promoting a favorable immune bias. (3) Protein Self-Assembling Vaccines: This category includes VLPs and cage-like proteins. VLPs are formed through the self-assembly of viral structural proteins fused with Aβ epitopes, maintaining immunogenicity while reducing toxicity. Cage-like proteins form nanoscale cage structures that encapsulate Aβ oligomers. (4) Liposomal Vaccines: Liposomes are composed of materials similar to human cell membranes. Liposomal vaccines anchor Aβ epitopes onto the surface of these lipid vesicles. This strategy can reduce Aβ deposition without inducing a significant inflammatory response. (5) Bacteriophage Display Vaccines: These vaccines display Aβ epitopes on the surface of bacteriophages, effectively inducing a specific antibody response. (6) Mucosal Vaccines and Novel Adjuvants: Mucosal vaccines administer antigens via mucosal routes. They utilize novel adjuvants to activate innate immunity, thereby triggering a specific immune response. The required immunization dose for this route is often lower than that needed for subcutaneous injection. These diverse vaccine presentation strategies enrich the field of AD vaccine research and offer multiple potential pathways to address safety concerns and enhance immune efficacy.

In the effort to develop vaccines, beyond the form of the antigen itself, the vaccine delivery or display system represents another significant and promising area for innovation. Improving and innovating vaccine delivery or display systems can not only enhance vaccine efficacy and safety but also, when combined with novel antigen formats, facilitate the production of more potent vaccine products. The following is an introduction to several innovative vaccine delivery or display systems (Figure 3).

#### 4.2.1 Dendritic cell vaccines

The preparation of dendritic cells (DCs) vaccines primarily involves the *ex vivo* “stimulation” of a patient’s DCs with Aβ protein. Following administration to the patient, these activated DC can elicit a specific immune response, leading to the generation of antibodies and thereby achieving the clearance of Aβ amyloid protein. Luo et al. (2012) developed a DC-based vaccine by co-culturing DC with a mutant form of Aβ1-42. Experimental mice were then immunized exclusively with the DC vaccine sensitized with the mutant Aβ1-42 peptide. In comparison to the control group, which received the wild-type Aβ1-42 peptide emulsified in Freund’s adjuvant, mice that received the mutant Aβ1-42 peptide-sensitized DC vaccine demonstrated elevated serum antibody levels, restored cognitive function, and a marked reduction in Aβ accumulation, all in the absence of a Th1-type inflammatory response. DC vaccines activate the immune system through a relatively mild mechanism, maintaining a balanced immune response that enables the clearance of Aβ while avoiding the induction of inflammation. Their demonstrated efficacy and favorable safety profile have established them as a broadly promising and innovative vaccine delivery platform for the development of active immunotherapies against AD.

#### 4.2.2 Viral vector vaccines

Viral vector vaccines utilize recombinant viruses as gene delivery tools to introduce the Aβ gene into host cells. This enables sustained expression of Aβ antigens *in vivo*, thereby inducing an immune response. Commonly employed viral vectors include adeno-associated virus (AAV), adenovirus, and influenza virus, among others. Zhang et al. (2003) employed an adenoviral vector encoding Aβ1-42 and the cholera toxin B subunit to prepare recombinant viruses. These were administered to transgenic AD model mice via intramuscular, mucosal, and oral routes for immunization. Results demonstrate that this vaccine can induce anti-Aβ antibody production, improve cognitive function, and significantly reduce amyloid plaques within the brain.

Similarly, Hara et al. (2004) developed an oral AD vaccine using an adenoviral vector encoding Aβ1-42 and Aβ1-21. Oral vaccines offer distinct advantages: they require no adjuvant, the intestinal immune system effectively suppresses Th1-type immune responses while promoting Th2-type immune responses, and they feature safe and convenient administration. The adenoviral vector vaccine constructed by Zou et al. (2008) expresses 4Aβ1-15 and incorporates the molecular adjuvant GM-CSF, further enhancing the vaccine’s immunological efficacy. The primary advantages of viral vector vaccines include their well-defined genetic structure, ease of recombinant modification, high infection efficiency, capacity for sustained expression of target genes, and the targeting specificity exhibited by certain vectors. However, safety concerns remain significant factors limiting viral vector applications, particularly since risks associated with viral infection and cellular transformation cannot be completely eliminated.

#### 4.2.3 Protein self-assembling vaccine

Due to safety concerns associated with recombinant viral vector vaccines, some researchers have begun to focus on the research and development of self-assembling protein vaccines. The vectors of such vaccines are mainly divided into two categories: VLPs and non - viral cage - like protein particles (CPs).

VLPs are self-assembling virus-derived protein assemblies that are spatially analogous to natural viruses. While retaining potent immunogenicity in the human body, they have reduced toxic effects, thus serving as outstanding vaccine vectors. Feng et al. (2013) fused the gene encoding the hepatitis B virus core antigen with the genes of two fragments corresponding to the N-terminal 15 amino acids (Sol et al., 2023) of lysine-linked Aβ, and then introduced the fused gene into *Escherichia coli*. Subsequently, the C2Aβ15C fusion protein was expressed and obtained through incubation. This C2Aβ15C protein is capable of self-assembling into Aβ-HBc VLPs. When BALB/c mice were immunized with these VLPs, it was observed that the serum from the immunized mice could inhibit the aggregation of Aβ fibrils, and no T-cell response to Aβ was induced.

CPs are nanoscale cage structures self-assembled from protein monomers; common protein monomers include, but are not limited to, ferritin, VLPs and chaperonins. Maity et al. (2024) genetically fused the Aβ peptide with ferritin monomers, then introduced the fused product into *Escherichia coli*, and obtained the purified Fr-Aβ42 monomer. This monomer can remain stable at 85 °C and efficiently encapsulate Aβ oligomers, which has laid a crucial foundation for the subsequent development of CPs vaccines for the treatment of AD.

#### 4.2.4 Liposomal vaccines

Liposomes are hollow vesicles formed by lipids, whose composition is similar to that of human cell membranes. Liposome-based vaccines utilize liposomal nanoparticles as carriers to deliver specific antigen fragments to the immune system, thereby activating the body’s own capacity to generate targeted immune responses, which in turn clear toxic protein aggregates from the brain. Nicolau et al. (2002) pioneered the development of a vaccine by incorporating the Aβ1–16 peptide sequence into liposomes and administering it to NORBA transgenic mice. Their study demonstrated that this liposomal vaccine effectively prevented the formation of amyloid plaques in young mice and significantly reduced pre-existing plaques in aged mice. This groundbreaking research established the feasibility of liposome-based Aβ vaccines. Building upon this foundational research, Muhs et al. (2007) conjugated palmitoylated lysine to both ends of the antigen Aβ 1-15, enabling it to anchor on the surface of liposomes, thus developing the ACI-24 vaccine which was then administered to the AP-PxPS1 mouse model. The experimental results showed that the insoluble, plaque-associated Aβ1-40 and Aβ1-42 in mice were significantly reduced, the soluble Aβ1-42 was also significantly decreased, while the soluble Aβ1-40 only showed a downward trend, with no inflammatory response observed. In subsequent clinical trials, ACI-24 demonstrated a favorable safety profile and pharmacodynamic response in patients with sporadic AD. Consequently, AC Immune company optimized ACI-24 based on these findings, leading to the development of ACI-24.060, which is currently being evaluated in clinical trials (Sol et al., 2023). As a fully chemically synthesized nano-delivery system, liposomal vaccines not only enable efficient delivery and presentation of multiple antigenic epitopes but also circumvent the potential risk of insertional mutagenesis associated with viral vectors. This positions them as a promising platform for vaccine presentation with significant developmental potential.

#### 4.2.5 Bacteriophage display vaccines

Phage display vaccines are developed through the genetic modification of bacteriophages, which are non-pathogenic to humans, enabling them to display target antigen fragments on their surface coat. Upon introduction into the human body, these phage particles are taken up and processed by antigen-presenting cells. The resulting antigen fragments are subsequently presented to immune cells, thereby efficiently inducing specific humoral and cellular immune responses against the target antigen. Frenkel et al. (2003) displayed the Aβ_3-6_ epitope (EFRH) on the surface of filamentous bacteriophages. Immunization of APP transgenic mice with this recombinant bacteriophage resulted in a significant reduction of Aβ deposition in the brain. Bacteriophage display technology enables precise control over the presentation mode of antigenic epitopes, facilitating the induction of more specific antibody responses.

#### 4.2.6 Mucosal vaccines and novel adjuvants

Mucosal vaccines, following antigen administration via the mucosal route, utilize novel adjuvants to activate innate immunity, thereby eliciting both humoral and cellular immune responses. This leads to the precise clearance of β-amyloid plaques in the brain and ultimately results in improved cognitive function. Lemere et al. (2001) administered vaccines intranasally to PDAPP mice, achieving a 50%–60% reduction in cerebral Aβ deposition. By incorporating *E. coli* heat-labile toxin (LT) and its detoxified mutant LT (R192G) as mucosal adjuvants in the intranasal vaccine formulation, they observed a 12-fold and 16-fold increase in induced Aβ-specific antibody titers, respectively, compared with adjuvant-free formulations. Frenkel et al. (2005) proposed a novel intranasal vaccine against AD. Immunization with a combination of glatiramer acetate and the proteosome-based adjuvant IVX-908 successfully cleared β-amyloid from the brains of mouse models, resulting in a significant 83% reduction in cerebral amyloid fibril levels compared to the control group. They also observed that both subcutaneous and intranasal vaccination were associated with microglial activation and an increase in T-cell numbers. Mucosal vaccination not only requires a lower immunization dosage than subcutaneous administration but also offers greater convenience for future clinical applications. These diversified vaccine strategies have enriched the AD vaccine research field, providing multiple potential approaches to address safety concerns and enhance immunological efficacy.

## 5 Challenges and prospects

Despite significant progress in AD vaccine research, numerous challenges remain. Addressing these challenges will constitute the primary focus of future studies. Whether antibodies induced by vaccines or passively administered therapeutic antibodies, all must traverse the BBB to bind with Aβ deposition in the brain and exert their effects. However, the BBB plays a protective role for the brain, limiting the entry of large molecules such as antibodies. Studies indicate that only minimal amounts of antibodies can cross the BBB under normal physiological conditions (Greenberg et al., 2003). This may partially explain why certain clinical trials showed limited therapeutic efficacy despite high serum antibody levels. Several potential solutions exist: first, leveraging the mild and localized inflammatory response potentially induced by Aβ immunotherapy itself to temporarily enhance BBB permeability; second, developing specialized technologies to facilitate antibody transport across the BBB, such as utilizing receptor-mediated transcytosis systems; An ideal AD vaccine should induce sufficiently strong humoral immune responses to generate high levels of anti-Aβ antibodies while avoiding robust cellular immune responses, particularly Th1-type responses. However, the immune system constitutes a complex network where precisely controlling the type and intensity of immune responses remains challenging. Current research strategies primarily focus on: 1) using truncated Aβ fragments devoid of T-cell epitopes; 2) selecting appropriate adjuvants to favor Th2-type responses; Utilizing DNA vaccines or viral vector vaccines to achieve sustained antigen expression, thereby inducing mild yet durable immune responses; optimizing immunization protocols including dosage, intervals, and administration routes. AD is a slowly progressive neurodegenerative disorder, where pathological changes may begin 10–20 years before clinical symptoms manifest. Consequently, the timing of vaccine intervention may be critically important. Animal studies demonstrate that preventive immunization prior to Aβ deposition yields optimal immunological efficacy, whereas therapeutic effects remain relatively limited in animals with substantial existing deposits. This implies that AD vaccines may be more suitable for prevention or early-stage patients with mild symptoms, rather than those in the middle or advanced stages of the disease. Future research needs to determine the optimal therapeutic time window, which may require early screening and intervention in high-risk populations.

The pathological changes in AD include not only Aβ deposition but also NFTs formed by hyperphosphorylated tau protein, neuroinflammation, synaptic loss, and neuronal death. Studies indicate that Aβ immunotherapy may also influence tau pathology. For instance, Oddo et al. (2004) found in the 3xTg-AD mouse model that Aβ immunization not only cleared Aβ deposits but also reduced hyperphosphorylated tau protein. However, the mechanism and universality of this effect require further investigation. Furthermore, evidence indicates that Aβ oligomers, rather than fibrils or monomers, constitute the primary toxic substance responsible for AD pathogenesis (Walsh et al., 2002). Therefore, an ideal vaccine should specifically target these toxic oligomers without affecting Aβ monomers that may possess physiological functions.

### 5.1 Future research directions

Based on current progress and challenges in AD vaccine research, future studies may focus on the following directions:

With growing recognition of AD heterogeneity, future strategies may include developing personalized vaccines tailored to distinct AD subtypes or genetic backgrounds (e.g., ApoE genotypes). For instance, carriers of the ApoE ε4 allele may exhibit differential responses to immunotherapy, necessitating adjustments in vaccine formulation or dosage. Given the complexity of AD’s pathological mechanisms, single-target therapies may prove insufficient to halt disease progression. Future research may explore combination therapies integrating vaccines with other treatments, such as anti-tau therapies, neuroprotective agents, or anti-inflammatory medications. For example, concurrent use of NSAIDs during vaccine administration may prevent or mitigate immunotherapy-induced inflammatory responses.

Beyond Aβ, other AD-associated proteins like tau may also serve as vaccine targets. Currently, several vaccines targeting the tau protein are in clinical development. For instance, ACI-35.030 (Sol et al., 2025) is in a Phase 2b trial, and AADvac1 (Kovacech et al., 2024) has completed Phase 2 clinical trials, with preparations underway for a larger-scale trial. The development of multivalent vaccines simultaneously targeting both Aβ and tau proteins is currently being actively explored, aiming to more comprehensively intervene in the AD pathological process. With continuous advancements in vaccine technology, novel platforms such as mRNA vaccines and virus-like particle vaccines are being explored for AD treatment. These emerging technologies may offer safer and more effective antigen presentation methods, enabling precise modulation of immune responses. Optimizing immunization protocols—including delivery routes (intramuscular injection, subcutaneous injection, intranasal administration, oral delivery, etc.), dosage, immunization intervals, and booster strategies—is crucial for enhancing vaccine efficacy and safety. For example, the “prime-boost” strategy (e.g., DNA vaccine priming followed by protein vaccine boosting) has been demonstrated to significantly enhance immune responses (Davtyan et al., 2010).

## 6 Conclusion

AD vaccine research has evolved from initial optimism through setbacks to a phase of diversified development. Although the first-generation vaccine AN-1792 failed in clinical trials due to safety concerns, it provided valuable experience that has propelled the advancement of next-generation vaccines. Currently, multiple vaccine candidates based on different strategies are at various developmental stages, including short-peptide vaccines, DNA vaccines, and viral vector vaccines. These vaccines aim to induce protective antibody responses while minimizing adverse T-cell responses. Challenges in AD vaccine research include limitations imposed by the BBB, precise modulation of immune responses, determination of therapeutic time windows, and interactions with other pathological changes. Addressing these challenges requires multidisciplinary collaboration and sustained research efforts.

Although the path ahead remains lengthy, AD vaccines—as a strategy directly targeting the core pathology of the disease—still hold immense potential and promising prospects. As our understanding of AD pathogenesis deepens and vaccine technology advances, we believe AD vaccines will likely become effective preventive and therapeutic tools against this devastating disease in the near future, offering hope to millions of patients and their families.
